# Vaccination coverage and factors associated with adherence to the vaccination schedule in young children of a rural area in Burkina Faso

**DOI:** 10.1080/16549716.2017.1399749

**Published:** 2017-11-29

**Authors:** Moubassira Kagoné, Maurice Yé, Eric Nébié, Ali Sie, Anja Schoeps, Heiko Becher, Olaf Muller, Ane Baerent Fisker

**Affiliations:** ^a^ Ministry of Health, Centre de Recherche en Santé de Nouna, Nouna, Burkina Faso; ^b^ Institute of Public Health, Medical School, Ruprecht-Karls-University, Heidelberg, Germany; ^c^ Institut für medizinische Biometrie und Epidemiologie, Universitätsklinikum Hamburg-Eppendorf, Hamburg, Germany; ^d^ Research Centre for Vitamins and Vaccines, Bandim Health Project, Statens Serum Institut, Copenhagen, Denmark; ^e^ Bandim Health Project, Bissau, Guinea-Bissau

**Keywords:** Health and demographic surveillance systems, vaccination coverage, risk factors, vaccination schedule, expended programme on immunization

## Abstract

**Background**: Vaccination is an important tool for reducing infectious disease morbidity and mortality. In the past, less than 80% of children 12–23 months of age were fully immunized in Burkina Faso.

**Objectives**: To describe coverage and assess factors associated with adherence to the vaccination schedule in rural area Burkina Faso.

**Methods**: The study population was extracted from the Nouna Health and Demographic surveillance system cohort. Data from four rounds of interviews conducted between November 2012 and June 2014 were considered. This study included 4016 children aged 12–23 months. We assessed the effects of several background factors, including sex, factors reflecting access to health care (residence, place of birth), and maternal factors (age, education, marital status), on being fully immunized defined as having received *Bacillus* Calmette–Guérin (BCG), three doses of diphtheria–tetanus–pertussis and oral polio vaccine, and measles vaccine by 12 months of age. The associations were studied using binomial regression to derive prevalence ratios (PRs) in univariate and multivariate regression models.

**Results**: The full vaccination coverage increased significantly over time (72% in 2012, 79% in 2013, and 81% in 2014, *p* = 0.003), and the coverage was significantly lower in urban than in rural areas (PR 0.84; 0.80–0.89). Vaccination coverage was neither influenced by sex nor influenced by place of birth or by maternal factors.

**Conclusion**: The study documented a further improvement in full vaccination coverage in Burkina Faso in recent years and better vaccination coverage in rural than in urban areas. The organization of healthcare systems with systematic outreach activities in the rural areas may explain the difference between rural and urban areas.

## Background

Childhood vaccination has contributed to major global reductions in morbidity and mortality [1,2] and is considered to be the most successful public health intervention in terms of number of deaths averted per year [3,4]. However, the World Health Organization (WHO) and the United Nations Children’s Fund (UNICEF) estimate that 1.5 million children worldwide continue to die from vaccine-preventable diseases every year because of sub-optimal vaccination coverage [5]. The largest number of these deaths occurs in Sub-Saharan Africa (SSA) and South-East Asia [6] where more than 70% of unvaccinated children worldwide live [4]. This emphasizes the particular need for continued monitoring of vaccination programme performance to detect potential problems and to identify appropriate solutions in these regions.

All the African countries are implementing the WHO Expanded Programme on Immunization (EPI). The immunizations in the EPI include antigens against tuberculosis, diphtheria, tetanus, and pertussis (DTP), polio, and measles, and confer protection against tetanus for newborn children and their mothers through vaccination of pregnant women. In some countries, other vaccines (e.g. against hepatitis B, *Haemophilus influenzae* type B, pneumococcus, rotavirus, or yellow fever) are included in the programme [7]. The aim of the routine immunization program is to deliver a complete number of antigens in a timely, safe, and effective way to all children and women.

A well-performing vaccination programme should obtain high and equitable coverage for all antigens. The Global Vaccine Action Plan specified 2015 targets as 90% national coverage with DTP3 and coverage of at least 80% in all districts worldwide [8]. However, many countries still fall short of these targets [9]. Research has been undertaken in a number of SSA countries to identify the determinants of childhood vaccination coverage [10–15]. Factors that commonly affect vaccination programme performance are either health-system-related (e.g. quality of services, distance, security, waiting time, reliability of vaccination session schedule) or user-related (e.g. family characteristics, parental attitudes and knowledge) [16,17].

Substantial efforts have been made to strengthen the immunization program in Burkina Faso. Since 1980, community nurses in all major villages have provided vaccines and are involved in outreach vaccination sessions [18]. Immunization service performance is monitored by coverage indicators for DTP3 or individual antigens by 12 months of age. This process considers only the number of vaccines administered, but not the age of the child at the time of vaccination and the adherence to the vaccination schedule. When assessing vaccination coverage, the focus has mainly been on DTP3 coverage by 12 months of age [8].

Monitoring based on coverage at 12 months of age does not reflect all shortcomings in the implementation of the vaccination policy. Vaccines are often given later than scheduled partly because of the ‘restrictive vial-opening policy’. In Guinea-Bissau, focus on not wasting vaccines has led to a policy of not opening *Bacillus* Calmette–Guérin (BCG) and measles vaccine (MV) vials unless several children are present to be vaccinated [19,20], and similar policies are implemented in Burkina Faso. This policy creates missed vaccination opportunities for many children and leads to delayed vaccination with BCG and MV. Delays may render children unprotected against infections for extended periods and cause changes in the vaccination sequence. Changes in sequences may be important if vaccines in addition to the specific effects alter the susceptibility to other infections; for example, DTP with or after MV seems to be associated with a higher mortality than MV after DTP [21]. Such patterns are not discovered if merely coverage by 12 months is presented.

We assessed vaccination coverage and factors associated with being fully immunized by 12 months of age in children aged 12–23 months. We also describe the timeliness of vaccination through assessing the proportion receiving vaccines in the recommended sequence and the median age of reception of a specific vaccine as well as the proportion of missed opportunities among vaccinated children.

## Methods

### Setting and study population

The study was conducted in the area of the Nouna Health and Demographic Surveillance System (HDSS). Nouna HDSS is located in the North West of Burkina Faso, in Nouna Health District 300 km from the capital Ouagadougou. The research area is 1775 km^2^ [22]. In 2014, the HDSS comprised about 100,000 inhabitants residing in Nouna town and the surrounding 58 villages. The predominant economic activity in the region is agriculture. The annual growth rate is 2.8% with a fertility rate of 6.2. Mortality in Nouna HDSS is high. The infant mortality for 2010 was 27/1000 live births, and the under-five mortality was 81/1000 live births [23]. The area has 16 peripherals health centres.

The study population for the present study was extracted from the HDSS cohort. Data from four rounds of interviews conducted between November 2012 and June 2014 were used. The study included children who were between 12 and 23 months old at the time of the visit, were alive, and had their vaccination card seen. If a child had more than one visit, the information obtained at the first visit was used.

### Vaccination services

The routine vaccination programme in Burkina Faso in 2010–2014 included five different vaccines for the prevention of nine pathogens: (1) BCG against tuberculosis, (2) Oral Polio Vaccine (OPV), (3) Pentavalent Vaccine against diphtheria, tetanus, pertussis, hepatitis B, and *Haemophilus influenzae* type b (Penta), (4) yellow fever vaccine, and (5) MV. The recommended vaccination schedule in Burkina Faso was BCG and first dose of OPV (OPV0) at birth; three doses of Penta and OPV at 8, 12, and 16 weeks; and measles and yellow fever vaccination at 9 months of age. Children living in villages with a peripheral health centre (CSPS) received routine vaccinations during monthly vaccination sessions. The CSPS also arranged vaccination sessions in villages in their catchment area once per month as well as catch-up sessions for those who missed the normal sessions at the CSPS or outreach days in the community.

The vaccines are provided through UNICEF and GAVI, and are free of charge for the parents. Vaccines are registered on a health card, which is given to the mother at her first antenatal care visit. The card is used for both the mother’s information during the pregnancy and for the child’s information, vaccinations, and birth weight of the child. Mothers who do not attend antenatal care are provided a vaccination card at the first vaccination contact for the child.

### Assessment of vaccination coverage

The collection of vaccination data took place at the regular vital-event registration rounds in the HDSS. The HDSS rounds collect routine data at 3 times/year visits from all households of the HDSS area. During these visits, vaccination data were collected from the vaccination cards for all children younger than 3 years of age. At all visits, the date of the visit and whether the child’s health card was seen were recorded. Provided the card was seen, the dates of all vaccines were noted on the child’s HDSS form. At a following round, the field assistant brought forms to the household of the individual child with preprinted dates of the vaccinations already registered. The field assistant inspected the card and updated the child’s vaccination information.

When a round finished in a village, the questionnaires were sent to the data-entry team. Data were entered in Microsoft Access 2007. Data-consistency checks were carried out during data entry where the system prompted the user if impossible values were entered (i.e. date of vaccination before date of birth). During data entry, questionnaires with missing or unclear information were sent back to the field supervisors and, if necessary, to the interviewers for correction. Following data entry, checks were made on consistency. Further validation was carried out through duplicate data entry of 5% of all questionnaires by the data-entry supervisor.

### Information on background factors

Information on background variables (maternal age, maternal education, place of birth, occupation, religion, marital status, season of birth, area of residence, and ethnic group) was collected during the HDSS rounds.

### Analyses

We determined the vaccination coverage of all antigens by 12 months of age and ages for median coverage as the age where 50% of children had received a particular vaccine. Vaccination status was determined based on the first visit to a child aged 12–23 months in 2012–2014 at which vaccination status was assessed by inspecting the vaccination card. The children who did not present a vaccination card at the time of the visit and the children who migrated or died before the visit were not included in the analysis. We compared the distribution of background factors for children who were included and children who were not included.

### Definitions

We defined a fully immunized child (FIC) as completion of the core EPI vaccinations by 12 months of age not including OPV0 at birth, since this vaccine is only given during the first weeks of life, and yellow fever vaccine, which is not globally recommended by WHO [7]. To describe adherence to the vaccination programme, we subdivided the FIC children into FIC in sequence (FICIS) which was defined as the WHO recommended sequence of vaccinations, i.e. BCG before OPV1, OPV1 = Penta1, OPV2 = Penta2, OPV3 = Penta3, and Penta3 before MV. If this sequence was violated, children were defined as FIC out of sequence (FICOS).

We further described the children missing one or more vaccines by the number of vaccines missing by 12 months of age and subdivided this into different antigens. Missed opportunities were defined as contacts with the health system where a vaccine dose could have been given, but where it was not received. For BCG, we defined a contact with the health system as either being born at a health facility or having received other vaccines but not BCG. Missed opportunities for later vaccines were time points where some, but not all, age-appropriate vaccines were given, i.e. children missing Penta3 and MV, who received only the one of the vaccines when seeking vaccination after 9 months of age.

### Statistical analysis

Our primary analysis focused on coverage and factors associated with being FIC versus not FIC. Associations between background factors and vaccination coverage were studied using binomial regression in univariate and multivariate regression models. A log-link function was used to obtain prevalence ratios (PR) using the command ‘binreg’ in Stata. The estimated PR and corresponding 95% confidence intervals are presented together with the *p*-values from the overall test (Wald) of no association between the factor and FIC.

Similarly, we assessed the factors associated with being FICIS among children who were FIC.

Data were analysed using STATA V.12.0.

### Ethical approval

This study was part of the OPTIMUNISE project, which was approved by the National Ethics Committee in Burkina Faso and by the local Ethical Committee in Nouna. Informed community consent was sought for the implementation of the additional survey questionnaire during routine HDSS procedures.

## Results

### Sample characteristics

Of the total of 6579 children visited between 12 and 23 months of age in the Nouna HDSS during the period of the study, 4273 (65%) had at least one visit with a card seen. We excluded 257 children because of data problems (e.g. vaccinated before birth or after the last visit), leaving 4016 children in the study. The distribution of included and excluded children did not differ by sex, maternal education, season of birth, and twinning status (Supplementary Table). However, there were slight but statistically significant differences for other background factors. More children were included in the later years, and the urban area made up 20% of the population in the study but only 17% among the excluded children. There were also fewer children born at home (8%) in our sample than among the excluded children (11%). Differences in distribution of ethnicity and religion were also seen (Supplementary Table).

Of the 4016 children under study, 3210 were resident in the rural area and 806 in the urban area. There were similar numbers of boys and girls included: 2021 were female, 1995 were male, and 93% of mothers in the study were married. A total of 87% of the children were delivered at a health facility.

### Vaccination status

Seventy-eight percent (3148/4016) of children were FIC by 12 months of age. The proportion of FIC increased over time, increasing from 72% in 2012 to 79% in 2013 and 81% in 2014, and the proportion did not differ between boys and girls (Table 1).

**Table 1.** Analyses of association between background factors and FIC^1^.

Factors	*N* (%^2^)	FIC (%)	PR (95%CI) ^3^	[*p*-value]	Adjusted PR^4^ (95%CI)	[*p*-value]
**Sex**				[*p* = 0.69]		[*p* = 0.85]
Male	1995 (50)	79	Ref.		Ref.	
Female	2021 (50)	78	0.99 (0.96–1.03)		1.00 (0.96–1.03)	
**Area**				[*p* < 0.001]		[*p* < 0.001]
Rural	3210 (80)	81	Ref.		Ref.	
Urban	806 (20)	68	0.84 (0.80–0.89)		0.83 (0.78–0.87)	
**Year of visit**				[*p* = 0.003]		[*p* < 0.001]
2012	648 (16)	72	Ref.		Ref.	
2013	2082 (52)	79	1.09 (1.03–1.15)		1.10 (1.04–1.16)	
2014	1286 (32)	81	1.12 (1.06–1.18)		1.11 (1.05–1.18)	
**Education**				[*p* = 0.50]		[*p* = 0.02]
Not educated	3646 (91)	78	Ref.		Ref.	
Educated	370 (9)	80	1.02 (0.97–1.08)		1.08 (1.02–1.13)	
**Place of birth**				[*p* = 0.86]		[*p* = 0.50]
Health facility	3506 (87)	79	Ref.		Ref.	
Home	337 (8)	79	0.99 (0.94–1.05)		0.98 (0.92–1.04)	
Missing	173 (4)	58				
**Occupation**				[*p* = 0.15]		Omitted
No salary	3656 (91)	80	Ref.			
Salary	186 (5)	75	0.94 (0.86–1.02)			
Missing	174 (4)	58				
**Ethnicity**				[*p* = 0.002]		Omitted
Bwamu	1098 (27)	79	Ref.			
Marka	1424 (35)	83	1.05 (1.01–1.09)			
Mossi	656 (16)	77	0.97 (0.92–1.02)			
Peulh	371 (9)	75	0.95 (0.89–1.02)			
Samo	223 (6)	71	0.90 (0.83–0.99)			
Others	75 (2)	80	1.01 (0.90–1.14)			
Missing	169 (4)	57				
**Religion**				[*p* = 0.65]		[*p* = 0.26]
Muslim	2354 (59)	79	Ref.		Ref.	
Catholic	1147 (29)	79	0.99 (0.95–1.03)		0.96 (0.93–1.00)	
Others	345 (9)	81	1.02 (0.96–1.08)		1.01 (0.94–1.08)	
Missing	170 (4)	58				
**Marital status**				[*p* = 0.21]		[*p* = 0.25]
Not married	125 (3)	74	Ref.		Ref.	
Married	3722 (93)	79	1.07 (0.96–1.19)		1.06 (0.96–1.18)	
Missing	169 (4)	57				
**Mother’s age**^5^				[*p* = 0.46]		[*p* = 0.34]
<19	622 (15)	77	Ref.		Ref.	
20–34	2631 (66)	80	1.03 (0.98–1.08)		1.03 (0.98–1.08)	
34–49	588 (15)	80	1.04 (0.98–1.10)		1.04 (0.98–1.10)	
Missing	175 (4)	58				
**Twin**				[*p* = 0.40]		Omitted
No	3729 (93)	79	Ref.			
Yes	118 (3)	82	1.04 (0.95–1.13)			
Missing	169 (4)	57				
**Season of birth**				[*p* = 0.69]		Omitted
Dry season	2379 (59)	79	Ref.			
Rainy season	1637 (41)	78	0.99 (0.96–1.03)			

^1^FIC: fully immunized child: having received the following recommended vaccinations by 12 months of age: BCG, 3 doses of oral polio vaccine, 3 doses of pentavalent vaccine and one dose of measles vaccine. ^2^Number and percentage of the 4016 children fulfilling the criteria, the proportion with missing information corresponds to the remaining fraction. ^3^PR: prevalence ratio estimated in a binomial regression model. ^4^Estimated among the 3836 children with complete information on included factors (96%: 3836/4016). Occupation, Ethnicity, Twinning status, and Season of birth omitted due to over-parameterization of the model. ^5^Age of the mother at the time of the birth of the child classified as in Sie et al. [23]. Using maternal age in quartiles did not change the conclusions: ≤21 years: Reference; 22–25 years: PR = 1.01 (0.96–1.06); 27–32 years: PR = 1.03 (0.98–1.07); ≥33 years: PR = 1.03 (0.98–1.08)

We assessed the association between FIC and a large number of background factors in uni- and multivariate models (Table 1). We found that place of residence was significantly associated with vaccination status, with fewer children in the urban area being FIC than in the rural area: 68% for the urban area and 81% for the rural area, PR = 0.84 (0.80–0.89). The year of visit (*p* = 0.003) and ethnic group (*p* = 0.002) were also associated with vaccination status. Maternal education was associated with higher FIC coverage though only in the adjusted model: PR = 1.08 (1.02–1.13) for formal education compared with no formal education. In contrast, marital status and age of the mother were not associated with FIC, and nor was place or season of birth (Table 1).

**Figure 1.**
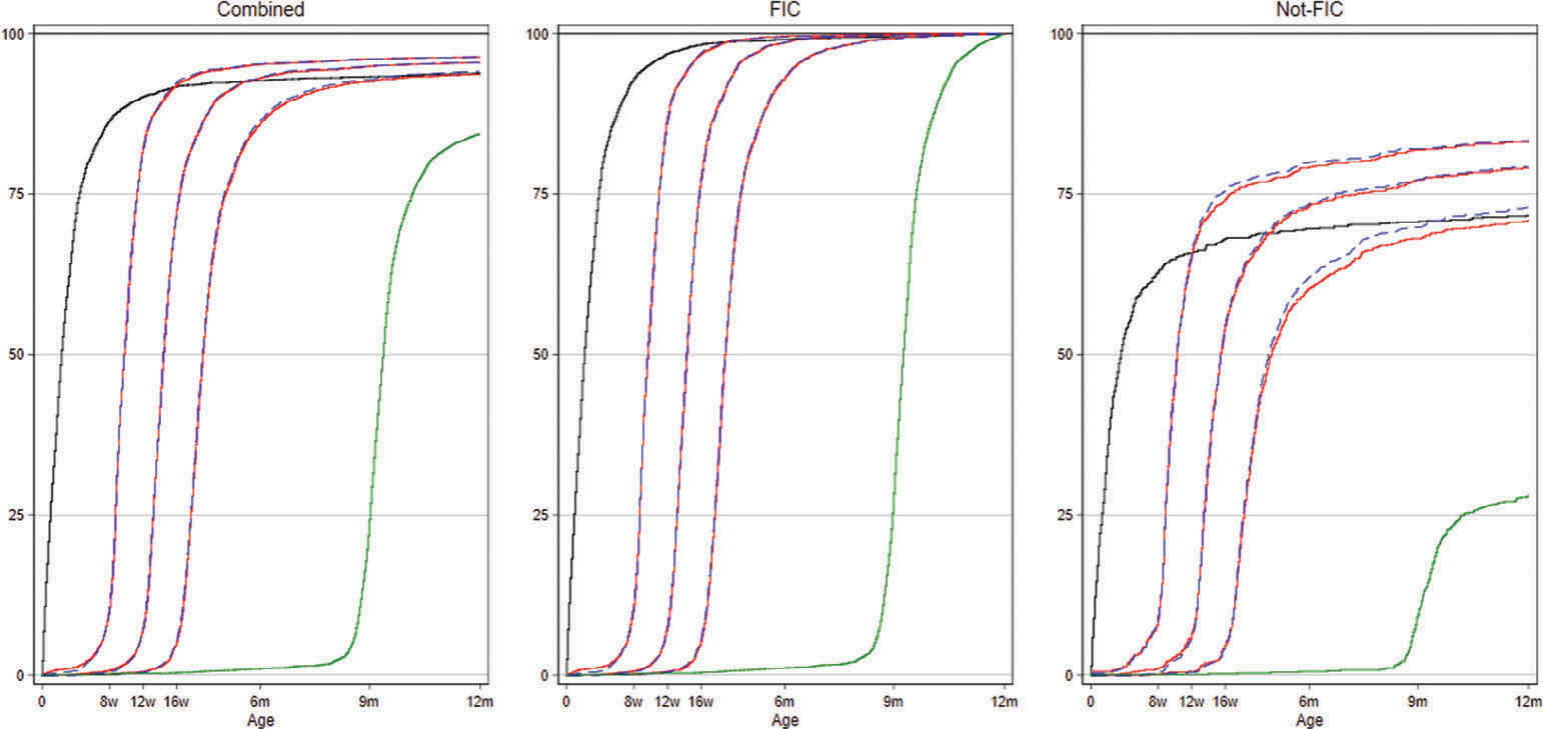
Vaccination coverage curves for children in Nouna HDSS 2012–2014 overall and subdivided by children who were fully immunized (FIC) and not FIC. Kaplan–Meier survival curves indicate vaccination coverage at specific ages. Black line: BCG vaccination; red lines: vaccination with pentavalent vaccines; blue lines: oral polio vaccine and green lines: measles vaccine.

We assessed vaccination coverage for the individual antigens and found that coverage by 12 months of age was very high (>94%) for the first vaccines (BCG, OPV1, and Penta1) and became low for the last vaccine, measles (84%, Figure 1).

### Adherence to the vaccination schedule

Eighty percent (2522/3148) of FIC children received all the vaccines in the recommended sequence (FIC in sequence, FICIS) and the proportion improved over the years (Table 2).

**Table 2.** Analyses of association between background factors and FICIS^1^ among FIC^2^.

Factors	*N* (%)^3^	FICIS (%)	PR (95%CI)^4^	[*p*-value]	Adjusted PR (95%CI)^5^	[*p*-value]
**Sex**				[*p* = 0.33]		[*p* = 0.58]
Male	1569 (50)	81	Ref.		Ref.	
Female	1579 (50)	79	0.98 (0.95–1.02)		0.99 (0.96–1.03)	
**Area**				[*p* = 0.39]		[*p* = 0.96]
Rural	2598 (83)	80	Ref.		Ref.	
Urban	550 (17)	82	1.02 (0.98–1.07)		1.00 (0.95–1.05)	
**Year of visit**				[*p* = 0.003]		[*p* < 0.001]
2012	468 (15)	76	Ref.		Ref.	
2013	1640 (52)	78	1.03 (0.98–1.09)		1.02 (0.96–1.09)	
2014	1040 (33)	85	1.12 (1.06–1.19)		1.12 (1.05–1.18)	
**Education**				[*p* = 0.00]		[*p* = 0.03]
Not educated	2853 (91)	79	Ref.		Ref.	
Educated	295 (9)	86	1.08 (1.03–1.14)		1.08 (1.02–1.14)	
**Place of birth**				[*p* = 0.86]		[*p* = 0.89]
Health facility	2782 (88)	80	Ref.		Ref.	
Home	266 (8)	80	0.99 (0.93–1.06)		1.00 (0.93–1.06)	
Missing	100 (3)	78				
**Occupation**				[*p* = 0.72]		Omitted
No salary	2908 (92)	80	Ref.			
Salary	139 (4)	81	1.02 (0.94–1.10)			
**Ethnicity**				[*p* = 0.29]		Omitted
Bwamu	868 (28)	80	Ref.			
Marka	1181 (38)	81	1.00 (0.96–1.05)			
Mossi	504 (16)	81	1.01 (0.96–1.06)			
Peulh	279 (9)	76	0.94 (0.87–1.01)			
Samo	159 (5)	78	0.97 (0.89–1.06)			
Others	60 (2)	87	1.08 (0.97–1.20)			
Missing	97 (3)	77				
**Religion**				[*p* = 0.34]		[*p* = 0.59]
Muslim	1869 (59)	80	Ref.		Ref.	
Catholic	902 (29)	82	1.03 (0.99–1.07)		0.96 (0.93–1.00)	
Others	279 (9)	79	0.99 (0.93–1.06)		1.01 (0.94–1.08)	
Missing	98 (3)	78				
**Marital status**				[*p* = 0.18]		[*p* = 0.25]
Not married	93 (3)	85	Ref.		Ref.	
Married	2958 (94)	80	0.94 (0.86–1.03)		1.06 (0.96–1.18)	
Missing	97 (3)	77				
**Mother’s age^5^**				[*p* = 0.32]		[*p* = 0.14]
<19	482 (15)	78	Ref.		Ref.	
20–34	2092 (66)	81	1.04 (0.99–1.10)		1.03 (0.98–1.08)	
34–49	472 (15)	80	1.02 (0.96–1.09)		1.04 (0.98–1.10)	
Missing	102 (3)	75				
**Twin**				[*p* = 0.85]		Omitted
No	2954 (94)	80	Ref.			
Yes	97 (3)	79	0.99 (0.89–1.10)			
Missing	97 (3)	77				
**Season of birth**				[*p* = 0.05]		Omitted
Dry season	1870 (59)	81	Ref.			
Rainy season	1278 (41)	78	0.96 (0.93–1.00)			

^1^FICIS: fully immunized child: having received the following recommended vaccinations by 12 months of age in the recommended sequence: BCG < oral polio vaccine (OPV)-1 + pentavalent (Penta)-1 < OPV2 + Penta2 < OPV3 + Penta3 < Measles vaccine. ^2^FIC: fully immunized child: having received the following recommended vaccinations by 12 months of age: BCG, 3 doses of oral polio vaccine, 3 doses of pentavalent vaccine and one dose of measles vaccine. ^3^Number and percentage of the 3148 children fulfilling the criteria, the proportion with missing information corresponds to the remaining fraction. ^4^PR: prevalence ratio estimated in a binomial regression model. ^5^Estimated among the 3042 children with complete information on included factors (3042/3148). Occupation, Ethnicity, Twinning status, and Season of birth omitted due to over-parameterization of the model. ^6^Age of the mother at the time of the birth of the child classified as in Sie et al. [23]. Using maternal age in quartiles did not change the conclusions: ≤21 years: Reference; 22–25 years: PR = 1.02 (0.97–1.07); 27–32 years: PR = 0.98 (0.93–1.03); ≥33 years: PR = 0.99 (0.94–1.04)

Regression analyses conducted on the FICIS children versus those with FICOS (i.e. the children missing one or more vaccines were excluded from these analyses) identified few factors associated with FICIS. In uni- and multivariate analyses, we found only year of visit and maternal education associated with FICIS, while being born in the rainy season was associated with lower FICIS in the univariate model (Table 2).

Nonadherence to the vaccination schedule can cause out-of-sequence vaccination among FIC children and children who do not to become FIC. Among non-FIC children, the majority (68% (586/868)) were missing only one vaccine, and for 72% (626/868) of these children, the missing vaccine was MV (Supplementary Figure). This is also reflected in the coverage curves (Figure 1).

We assessed the extent of missed opportunities for each vaccine. For BCG, 86% (2706/3148) of children who were FIC were not vaccinated at the first documented contact with the health system; among non-FIC children, this was seen for 84% (728/868). The recorded missed opportunities for OPV1 and Penta1 were 10% for FIC (327/3148) and 15% (128/868) for non-FIC. The recorded percentage of missed opportunities for MV is very low, <1% (9/3148) for FIC and 5% (47/868) for non-FIC children.

### Median vaccination coverage age

The age at which 50% of the children had been immunized with pentavalent vaccines was 68 days (12 days after the recommended age of 8 weeks) for the first dose, 101 days for the second dose (17 days after 12 weeks), and 135 days for the third dose (23 days after 16 weeks). The age at which 50% had received MV was 285 (15 days after recommended age of 9 months), and for BCG the median age was 16 days, though the vaccine should be given at birth. The median age for receiving different antigens did not change over time (data not shown).

## Discussion

### Main findings

The full immunization coverage increased over the study years and was higher in rural than in urban areas but did not differ by sex. Among the children who were fully vaccinated, 80% were vaccinated in the WHO recommended sequence of vaccination.

Lacking MV was the main cause of not being fully immunized, and delays in administration of the BCG could be reduced by vaccinating at first contact with the health facility.

### Consistency with other studies

The full immunization coverage for children aged 12–23 months reported from vaccination cards in this study was 78% with better coverage in the rural study area compared with the urban area. This supports findings from a former study conducted in the same study area. In September 2008 to December 2009, coverage in children aged 12–23 months was around 75%, with a significantly higher coverage in rural than in urban areas [24], and with more children receiving timely vaccines with the exception of BCG [25]. But our findings contrast with those from South Africa [26] and Mozambique [27] where increasing distances to health facilities were associated with lower coverage. This difference is likely attributable to the outreach vaccination teams functioning in the rural area, while mothers in the urban area have to seek vaccination in health facilities. In addition, the number of CSPSs increased in the rural area. Between 2009 and 2013, 233 new CSPSs were opened in Burkina Faso, an increase from 1373 in 2009 to 1606 in 2013 [28].

FIC coverage increased from 72% in 2012 to 81% in 2014. The coverage among the children included in our study is still lower than the national estimates: for the Nouna district, the administrative coverage was 107% in 2013, while the national coverage was 98% [28]. However, administrative coverage estimates are based on the number of vaccine doses administered to an estimated target population and very likely do not accurately reflect coverage [29].

Overall, 80% of children were vaccinated in sequence. Regression analyses show that only the year of visit, maternal education, and being born in the rainy season were associated with FICIS. That children born in the rainy season were less likely to be FICIS, while season of birth did not affect the proportion of FIC children, may indicate that weather conditions delayed some vaccination contacts, but not to the extent that coverage by 12 months was affected.

Not surprisingly, we found high vaccination coverage for vaccines recommended at birth and lowest coverage rates for vaccines given in late infancy but also longer delays for the first vaccine. This difference in coverage between these two sets of vaccines has also been documented in other studies in developing countries [30–32].

This study also reveals that the vast majority of children were not vaccinated with BCG on the first opportunity for vaccination in the Nouna health district. Other studies have also described similar patterns, and the shortage of vaccines and the unwillingness of health workers to open multi-dose vaccine vials if there are not enough children present for vaccination have been cited as reasons [33–35].

### Strengths and weaknesses

A major strength of this study is the set-up in the form of the health and demographic surveillance system covering a large proportion of the Nouna health district in Burkina Faso.

Data were collected through frequent home visits by experienced field workers and supervisors. The system that has been in place since 1993 [22] and the collection of vaccination data was piloted in 2008–2009 [24]. The precision of date of birth is high both due to HDSS and because most births occur in health facilities. Weaknesses include that children who died before the assessment age did not enter the coverage analysis and that we had to exclude a number of children due to incomplete data.

We used a seen vaccination card as the criterion to enter the study and did not rely on recall. This makes the age of vaccination accurate, but we cannot exclude the possibility that the children who did not enter our study due to having no seen card differ from those included. Due to the HDSS setting, we could compare background factors for included children and children who did not enter the study. While there were some smaller differences in background factors, these are not likely to explain the identified factors. In Guinea-Bissau, the coverage among children who did not have their card seen between 12 and 23 months but had a card inspected at a later occasion was slightly lower than among those who had a vaccination card seen between 12 and 23 months. We presume that the same pattern is true for our study setting. With a higher proportion of children from the source population included in the later years (Supplementary Table), we would expect that we include more children who are not vaccinated in our sample and therefore the estimated increase in coverage likely to be conservative.

### Interpretation and implications

Delays in vaccination potentially place children and their communities at an increased risk of serious infectious diseases. A previous study has shown that children under-vaccinated who had not received three or four doses of DTP vaccine were 18.56 (95% CI, 4.92–69.95) and 28.38 (95% CI, 3.19–252.63) times more likely, respectively, to have received a diagnosis of pertussis than children who were age-appropriately vaccinated [36]. While delays in DTP vaccination have an impact on pertussis immunity, the problem is not limited to DTP/penta. The low coverage of measles vaccination also indicates the need for strategies to address the situation.

The missing MV in our study puts the population at risk of measles epidemics. Furthermore, numerous studies have shown that MV is beneficial for child survival [21], and lacking an MV may therefore also be depriving children of a non-specific beneficial effect.

While the delay in BCG vaccination may not be important from a tuberculosis prevention perspective, it is likely to have marked effects on early child mortality. Previous studies have shown that BCG has beneficial non-specific or heterologous effects, providing protection also against many non-tuberculosis causes of death: in randomized trials of early BCG to low-birth-weight children, BCG at birth versus delayed BCG, as is normal practice, decreased neonatal mortality by 38% (17–54%) [37–39]. With most infant deaths occurring during the neonatal period [40] and with less than half of children vaccinated during the first two weeks of life, most children do not benefit from BCG when they are most at risk.

Our data indicate that to improve coverage and timeliness, avoiding missed opportunities for BCG vaccination is a low-hanging fruit, as 86% of children did not receive BCG at their first contact with the health centre. To improve FIC coverage, ensuring that all children receive MV is essential. We were not able to demonstrate that the low coverage is due to missed opportunities, as there were few other vaccination contacts after 9 months. However, with a system of community health workers in place as in Burkina Faso, emphasizing messages about MV could be attempted.

## Conclusion

In conclusion, place of residence year of visit and maternal education were the strongest factors influencing vaccination coverage and adherence to the vaccination schedule. Most children who did not complete the vaccination schedule were missing MV, and BCG vaccines were not given at the first opportunity. Avoiding missed opportunities, providing early BCG vaccination, and increasing the measles coverage could improve both child survival and immunization coverage.

## Supplementary Material

Supplemental Data

## Paper context

We assessed vaccination status and factors associated with different vaccination patterns among 4016 children aged 12–23 months, living in the Nouna Health and Demographic surveillance system area. Using data from four rounds of interviews between November 2012 and June 2014, we found that the full vaccination coverage increased significantly over time (from 72% to 81%, *p*=0.003). The coverage was significantly lower in urban than in rural areas (prevalence ratio 0.84; 0.80–0.89).

## References

[1] Plotkin S, Orenstein W, Offit PA. Vaccines. Vol. 5 Philadelphia: Saunders; 2008.

[2] Andre FE, Booy R, Bock HL, et al Vaccination greatly reduces disease, disability, death and inequity worldwide. Bull World Health Organ. 2008;86:140–9. 18297169 10.2471/BLT.07.040089 PMC2647387

[3] Tagbo BN, Uleanya ND, Nwokoye IC, et al Mothers’ knowledge, perception and practice of childhood immunization in Enugu. Niger J Paediatr. 2012;39.

[4] Duclos P, Okwo-Bele JM, Gacic-Dobo M, et al Global immunization: status, progress, challenges and future. BMC Int Health Hum Rights. 2009;9:S2. 19828060 10.1186/1472-698X-9-S1-S2 PMC2762311

[5] World Health Organisation Fact sheet: immunization coverage [cited 2017 10 04] Available from: http://www.who.int/mediacentre/factsheets/fs378/en/.

[6] Black RE, Cousens S, Johnson HL, et al Global, regional, and national causes of child mortality in 2008: a systematic analysis. Lancet. 2010;375:1969–1987. 20466419 10.1016/S0140-6736(10)60549-1

[7] World Health Organisation Vaccination recommendations. [cited 2017 Oct 04]. Available from: http://www.who.int/immunization/policy/Immunization_routine_table1.pdf

[8] World Health Organization Global Vaccine Action Plan 2011-2020. [cited 2017 Oct 04]. Available from: http://www.who.int/immunization/global_vaccine_action_plan/GVAP_doc_2011_2020/en/index.html.

[9] Subaiya S, Dumolard L, Lydon P, et al Global routine vaccination coverage, 2014. MMWR Morbidity and Mortality Weekly Report. 2015;64:1252–1255. 26562454 10.15585/mmwr.mm6444a5

[10] Fatiregun AA, Okoro AO Maternal determinants of complete child immunization among children aged 12-23 months in a southern district of Nigeria. Vaccine. 2012;30:730–736. 22137878 10.1016/j.vaccine.2011.11.082

[11] Sia D, Fournier P, Sondo BK [Local culture of vaccination: the role of central authorities in the health of the rural population in Burkino Faso]. Glob Health Promot. 2011;18:68–80. 21744670 10.1177/1757975911404747

[12] Babirye JN, Rutebemberwa E, Kiguli J, et al More support for mothers: a qualitative study on factors affecting immunisation behaviour in Kampala, Uganda. BMC Public Health. 2011;11:723. 21942999 10.1186/1471-2458-11-723 PMC3187758

[13] Wiysonge CS, Uthman OA, Ndumbe PM, et al Individual and contextual factors associated with low childhood immunisation coverage in sub-Saharan Africa: a multilevel analysis. PloS One. 2012;7:e37905. 22662247 10.1371/journal.pone.0037905 PMC3360654

[14] Hadler SC, Dietz V, Okwo-Bele JM, et al Immunization in developing countries In: Orenstein W, Offit PA, Plotkin S, editors. Vaccines. Philadelphia: Saunders Elsevier; 2008.

[15] Sanou A, Simboro S, Kouyate B, et al Assessment of factors associated with complete immunization coverage in children aged 12-23 months: a cross-sectional study in Nouna district, Burkina Faso. BMC Int Health Hum Rights. 2009;9:S10. 19828054 10.1186/1472-698X-9-S1-S10 PMC2762310

[16] Ojikutu RK Beliefs, knowledge and perception of parents to peadeatric vaccination in Lagos State, Nigeria. J Manag Sustainability. 2012;2.

[17] Sia D, Kobiane JF, Sondo BK, et al [Individual and environmental characteristics associated with immunization of children in rural areas in Burkina Faso: a multi-level analysis]. Sante. 2007;17:201–206. 18299262 10.1684/san.2007.0088

[18] Ministère de la santé, Secrétariat général, Direction générale de la santé, Direction de la prévention par les vaccinations Revue Approfondie du PEV 2009. Burkina Faso: 2010.

[19] Thysen SM, Byberg S, Pedersen M, et al BCG coverage and barriers to BCG vaccination in Guinea-Bissau: an observational study. BMC Public Health. 2014;14:1037. 25282475 10.1186/1471-2458-14-1037 PMC4195857

[20] Byberg S, Fisker AB, Rodrigues A, et al Household experience and costs of seeking measles vaccination in rural Guinea-Bissau. Trop Med Int Health. 2017;22:12–20. 27717100 10.1111/tmi.12793

[21] Higgins JP, Soares-Weiser K, Lopez-Lopez JA, et al Association of BCG, DTP, and measles containing vaccines with childhood mortality: systematic review. Bmj. 2016;355:i5170. 27737834 10.1136/bmj.i5170 PMC5063034

[22] Sie A, Louis VR, Gbangou A, et al The health and demographic surveillance system (HDSS) in Nouna, Burkina Faso, 1993-2007. Global Health Action. 2010;3:5284. 10.3402/gha.v3i0.5284 PMC2940452 20847837

[23] Sie A, Niamba L, Ye M, et al Rapport annuel: Système de surveillance démographique et de sante (SSDS) de Nouna CRSN. 2011.

[24] Ouedraogo N, Kagone M, Sie A, et al Immunization coverage in young children: a study nested into a health and demographic surveillance system in Burkina Faso. J Trop Pediatr. 2013;59:187–194. 23363884 10.1093/tropej/fms075

[25] Schoeps A, Ouedraogo N, Kagone M, et al Socio-demographic determinants of timely adherence to BCG, Penta3, measles, and complete vaccination schedule in Burkina Faso. Vaccine. 2013;32:96–102. 24183978 10.1016/j.vaccine.2013.10.063

[26] Fadnes LT, Jackson D, Engebretsen IM, et al Vaccination coverage and timeliness in three South African areas: a prospective study. BMC Public Health. 2011;11:404. 21619642 10.1186/1471-2458-11-404 PMC3126743

[27] Shemwell SA, Peratikos MB, Gonzalez-Calvo L, et al Determinants of full vaccination status in children aged 12-23 months in Gurue and Milange districts, Mozambique: results of a population-based cross-sectional survey. Int Health. 2017;9:234–242. 28810665 10.1093/inthealth/ihx020 PMC5881253

[28] Ministère de la Santé Annuaire statistique 2013. 2014.

[29] Burton A, Monasch R, Lautenbach B, et al WHO and UNICEF estimates of national infant immunization coverage: methods and processes. Bull World Health Organ. 2009;87:535–541. 19649368 10.2471/BLT.08.053819 PMC2704038

[30] Mutua MK, Kimani-Murage E, Ettarh RR Childhood vaccination in informal urban settlements in Nairobi, Kenya: who gets vaccinated? BMC Public Health. 2011;11:6. 21205306 10.1186/1471-2458-11-6 PMC3024932

[31] Breiman RF, Streatfield PK, Phelan M, et al Effect of infant immunisation on childhood mortality in rural Bangladesh: analysis of health and demographic surveillance data. Lancet. 2004;364:2204–2211. 15610807 10.1016/S0140-6736(04)17593-4

[32] Fisker AB, Hornshoj L, Rodrigues A, et al Effects of the introduction of new vaccines in Guinea-Bissau on vaccine coverage, vaccine timeliness, and child survival: an observational study. The Lancet Global Health. 2014;2:e478–e487. 25103521 10.1016/S2214-109X(14)70274-8

[33] Torun SD, Bakirci N Vaccination coverage and reasons for non-vaccination in a district of Istanbul. BMC Public Health. 2006;6:125. 16677375 10.1186/1471-2458-6-125 PMC1464125

[34] Agarwal S, Bhanot A, Goindi G Understanding and addressing childhood immunization coverage in urban slums. Indian Pediatr. 2005;42:653–663. 16085966

[35] Hutchins SS, Jansen HA, Robertson SE, et al Studies of missed opportunities for immunization in developing and industrialized countries. Bull World Health Organ. 1993;71:549–560. 8261558 PMC2393481

[36] Glanz JM, Narwaney KJ, Newcomer SR, et al Association between undervaccination with diphtheria, tetanus toxoids, and acellular pertussis (DTaP) vaccine and risk of pertussis infection in children 3 to 36 months of age. JAMA Pediatr. 2013;167:1060–1064. 24019039 10.1001/jamapediatrics.2013.2353

[37] Aaby P, Roth A, Ravn H, et al Randomized trial of BCG vaccination at birth to low-birth-weight children: beneficial nonspecific effects in the neonatal period? J Infect Dis. 2011;204:245–252. 21673035 10.1093/infdis/jir240

[38] Biering-Sørensen S, Aaby P, Lund N, et al Early BCG-Denmark and neonatal mortality among infants weighing &lt;2500 g: a randomized controlled trial. Clin Infect Dis. 2017;65:1183–1190. 10.1093/cid/cix525 PMC5849087 29579158

[39] Biering-Sorensen S, Aaby P, Napirna BM, et al Small randomized trial among low-birth-weight children receiving Bacillus Calmette-Gueerin vaccination at first health center contact. Pediatr Infect Dis J. 2012;31:306–308. 22189537 10.1097/INF.0b013e3182458289

[40] Lawn JE, Cousens S, Zupan J 4 million neonatal deaths: when? Where? Why? Lancet. 2005;365:891–900. 15752534 10.1016/S0140-6736(05)71048-5

