# Supplementary material for: Vaccination coverage and factors associated with adherence to the vaccination schedule in young children of a rural area in Burkina Faso
Source: Glob Health Action. 2017 Nov 29;10(1):1399749. doi: 10.1080/16549716.2017.1399749 (PMC5800485; doi:10.1080/16549716.2017.1399749)
Supplement: Supplemental Data [file ZGHA_A_1399749_SM1486.docx]

**Supplementary Figure: Among children not fully immunized, the proportion missing a specific vaccine and number of vaccines missing**


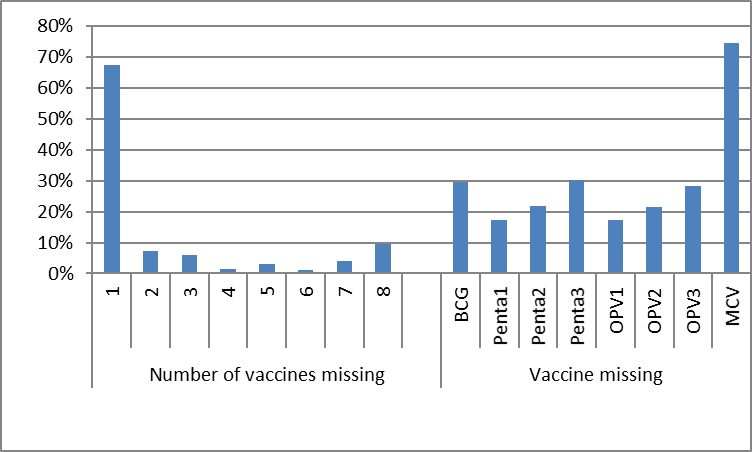


**Supplementary Table: Comparison of background factors for children included in and excluded from the analyses.**

| **Factors** | **Included** | **Excluded** | **P value, test of same distribution** |
| --- | --- | --- | --- |
| **Sex** | | |  |
| Male | 1995 (50) | 1271 (50) | 0.95 |
| Female | 2021 (50) | 1292 (50) |  |
| **Area** | | |  |
| Rural | 3210 (80) | 2121 (83) | 0.004 |
| Urban | 806 (20) | 442 (17) |  |
| **Year of visit** | | |  |
| 2012 | 648 (16) | 495 (19) | <0.001 |
| 2013 | 2082 (52) | 1490 (58) |  |
| 2014 | 1286 (32) | 578 (23) |  |
| **Education** | | |  |
| Not educated | 3646 (91) | 2338 (91) | 0.55 |
| Educated | 370 (9) | 225 (9) |  |
| **Place of birth** | | |  |
| Health facility | 3506 (87) | 2096 (82) | <0.001 |
| Home | 337 (8) | 285 (11) |  |
| Missing | 173 (4) | 182 (7) |  |
| **Occupation** | | |  |
| No salary | 3656 (91) | 2273 (89) | <0.001 |
| Salary | 186 (5) | 113 (4) |  |
| Missing | 174 (4) | 177 (7) |  |
| **Ethnicity** | | |  |
| Bwamu | 1098 (27) | 587 (23) | <0.001 |
| Marka | 1424 (35) | 1020 (40) |  |
| Mossi | 656 (16) | 347 (14) |  |
| Peulh | 371 (9) | 292 (11) |  |
| Samo | 223 (6) | 110 (4) |  |
| Others | 75 (2) | 38 (1) |  |
| Missing | 169 (4) | 169 (7) |  |
| **Religion** | | |  |
| Muslim | 2354 (59) | 1598 (62) | <0.001 |
| Catholic | 1147 (29) | 720 (28) |  |
| Others | 345 (9) | 76 (3) |  |
| Missing | 170 (4) | 169 (7) |  |
| **Marital status** | | |  |
| Not married | 125 (3) | 101 (4) | <0.001 |
| Married | 3722 (93) | 2292 (89) |  |
| Missing | 169 (4) | 170 (7) |  |
| **Mothers age** | | |  |
| <19 | 622 (15) | 408 (16) | 0.07 |
| 20 -34 | 2631 (66) | 1668 (65) |  |
| 34 -49 | 588 (15) | 317 (12) |  |
| Missing | 175 (4) | 170 (7) |  |
| **Twin** | | |  |
| No | 3729 (93) | 2313 (90) | 0.49 |
| Yes | 118 (3) | 81 (3) |  |
| Missing | 169 (4) | 169 (7) |  |
| **Season of birth** | | |  |
| Dry season | 2379 (59) | 1537 (60) | 0.56 |
| Rainy season | 1637 (41) | 1026 (40) |  |
